# Ulcerative Colitis Induces Changes on the Expression of the Endocannabinoid System in the Human Colonic Tissue

**DOI:** 10.1371/journal.pone.0006893

**Published:** 2009-09-04

**Authors:** Lucia Marquéz, Juan Suárez, Mar Iglesias, Francisco Javier Bermudez-Silva, Fernando Rodríguez de Fonseca, Montserrat Andreu

**Affiliations:** 1 Department of Gastroenterology, Hospital del Mar, Universidad Autónoma, Barcelona, Spain; 2 Laboratorio de Medicina Regenerativa, Fundación IMABIS, Málaga, Spain; 3 Department of Pathology, Hospital del Mar, Universidad Autónoma, Barcelona, Spain; Charité-Universitätsmedizin Berlin, Germany

## Abstract

**Background:**

Recent studies suggest potential roles of the endocannabinoid system in gastrointestinal inflammation. Although cannabinoid CB_2_ receptor expression is increased in inflammatory disorders, the presence and function of the remaining proteins of the endocannabinoid system in the colonic tissue is not well characterized.

**Methodology:**

Cannabinoid CB_1_ and CB_2_ receptors, the enzymes for endocannabinoid biosynthesis DAGLα, DAGLβ and NAPE-PLD, and the endocannabinoid-degradating enzymes FAAH and MAGL were analysed in both acute untreated active ulcerative pancolitis and treated quiescent patients in comparison with healthy human colonic tissue by immunocytochemistry. Analyses were carried out according to clinical criteria, taking into account the severity at onset and treatment received.

**Principal Findings:**

Western blot and immunocytochemistry indicated that the endocannabinoid system is present in the colonic tissue, but it shows a differential distribution in epithelium, lamina propria, smooth muscle and enteric plexi. Quantification of epithelial immunoreactivity showed an increase of CB_2_ receptor, DAGLα and MAGL expression, mainly in mild and moderate pancolitis patients. In contrast, NAPE-PLD expression decreased in moderate and severe pancolitis patients. During quiescent pancolitis, CB_1_, CB_2_ and DAGLα expression dropped, while NAPE-PLD expression rose, mainly in patients treated with 5-ASA or 5-ASA+corticosteroids. The number of immune cells containing MAGL and FAAH in the lamina propria increased in acute pancolitis patients, but dropped after treatment.

**Conclusions:**

Endocannabinoids signaling pathway, through CB_2_ receptor, may reduce colitis-associated inflammation suggesting a potential drugable target for the treatment of inflammatory bowel diseases.

## Introduction

The endocannabinoid system (ECS) has been described in the gastrointestinal tract in the epithelial, immune and neural compartments. It is involved in many physiological and physiopathological actions (peristalsis/contraction, secretion, gastric emptying, emesis, satiety and immunomodulation/inflammation and pain).[1]–[6] ECS roles comprise main facets of the pathogenesis of Inflammatory Bowel Disease (IBD) in humans, a disease that is likely to result from multiple factors, especially a disregulation of intestinal immune system and an inappropriate response to comensal bacteria or other luminal antigens.[7]–[9]

Components of ECS include cannabinoid CB_1_ and CB_2_ receptors, their endogenous lipid ligands (2-arachidony glycerol–2-AG; anandamide - AEA) and enzymes involved in their biosynthesis and release (DAGLα and DAGLβ for 2-AG; NAPE-PLD for AEA)[10]–[15], as well as mechanisms for cellular uptake and degradation, such as fatty acid amide hydrolase (FAAH) for AEA and monoacylglycerol lipase (MAGL) for 2-AG.[16], [17] The role of endocannabinoids and its derivatives in IBD is not completely known[18]–[22], although cannabinoid CB_1_ receptors have been proposed to participate in the epithelial wound healing during intestinal inflammation.[1]–[4], [20] Additionally, cannabinoid CB_2_ receptors are expressed in intestinal lamina propria suggesting a role in immunomodulation.[19], [20], [22]

Data from animal model and human studies have suggested an upregulation of the ECS in inflammation processes either by increased receptor expression or by an enhancement of endocannabinoid production.[23]–[27] Treatment with CB_1_ agonists, FAAH antagonists, inhibitors of endocannabinoid membrane transport, or genetic ablation of FAAH reduced inflammation.[23], [25], [28] Additionally, cannabinoid CB_2_ agonists cause inhibition of proinflammatory citokines such as tumoral necrosis factor alfa (TNFα) and IL8.[29] Thus, ECS is positioned to exert a protective role in many of the points where homeostasis breaks in IBD, although this antiinflammatory role of the ECS remains to be conclusively determined in humans.[25], [30]

The aim of the present study is to analyse, by immunocytochemistry, the expression of components of the endocannabinoid system such as cannabinoid CB_1_ and CB_2_ receptors and the enzymes involved in cannabinoid degradation (FAAH and MAGL) and biosynthesis (DAGLα, DAGLβ and NAPE-PLD), in normal human colonic tissue in comparison with untreated active ulcerative pancolitis at disease onset and after achieving remission, according to clinic and endoscopic criteria, and depending on severity of flare and treatment received.

## Methods

### Ethics statement

Biopsies and colonic resection samples were obtained after a written inform consent from all the patients, as requested by the clinical guides of Hospital del Mar. Research procedures were approved by the Hospital del Mar Clinical Research and Ethics Committee and were conducted according to the principles expressed in the Declaration of Helsinki.

### Subjects

Human colonic endoscopic biopsies were selected from 24 patients with a first ever flare of extensive Ulcerative Colitis (UC) diagnosed by clinical, endoscopic and pathological criteria (E3, Montreal classification).[31] In each patient rectal mucosal samples were obtained at onset, at first colonoscopy, before any treatment (acute group) and after achieving clinical (Truelove and Witts score <6 points)[32] and endoscopic remission (Mayo clinic score 0)[33], (quiescent group).

Twenty-two rectal samples were removed from colonic tissue of patients underwent colonic resections for colorectal cancer, at least 10 cm from the tumour (control group). In the control group, we confirmed histopathologically the absence of microscopic alterations. The analysis of the immunostaining patterns was carried out at transmural planes of the normal colonic tissue by comparing it with H&E staining.

Colonic samples were retrieved from tissue bank of Pathology Service at the Hospital del Mar from Barcelona, Spain. Data from each patient were collected retrospectively from medical records including age, sex, smoke and alcohol history, Body Mass Index (BMI) and comorbidity. In UC patients we recorded date of diagnosis, disease location (Montreal classification), endoscopic (Mayo clinic score) and clinical score (Truelove and Witts score: mild, moderate and severe) at onset, histological features and treatments received since initial diagnostic (5-aminosalicilates (5-ASA); corticosteroids; and/or the immunomodulators (CyclosporineA and/or Azathioprine). Table 1 shows some of these records that characterize each UC patients.

**Table 1:** Clinical characteristics of UC patients^1^.

Patient UC N°	Age	Sex	Smoker	BMI	Clinic Score	Endosc. Score	Histol. Score	UC Treatment
1	35	W	No	24,97	Moderate	2	Mild	5-ASA + cortic
2	29	W	No	26,1	Moderate	2	Moderate-Severe	5-ASA + cortic + IMM
3	29	M	15 cig/day	21,88	Severe	3	Moderate	5-ASA + cortic + IMM
4	28	M	Smoker	30,86	Moderate	2	Moderate	5-ASA + cortic
5	46	M	30 cig/day	28	Moderate	3	Severe	5- ASA + cortic
6	38	W	No	23,87	Mild	1	Severe	5-ASA
7	69	M	Ex-smoker	22	Mild	1	Mild	5-ASA + cortic
8	20	M	No	22,98	Severe	2	Mild	5-ASA + cortic + IMM
9	23	M	No	25,01	Moderate	2	Moderate	5-ASA + cortic + IMM
10	26	W	6 cig/day	23,42	Severe	3	Moderate-Severe	5-ASA + cortic
11	37	M	No	22	Mild	1	Mild	5-ASA
12	48	M	No	21,24	Moderate	2	Severe	5-ASA + cortic
13	34	M	Ex-smoker	22,86	Severe	2	Severe	5-ASA + cortic
14	61	M	Ex-smoker	23,26	Severe	2	Mild-moderate	5-ASA + cortic
15	28	W	No	23,05	Mild	2	Moderate	5-ASA
16	26	M	No	24,3	Moderate	2	Mild	5-ASA + cortic
17	39	M	No	22,52	Moderate	2	Mild	5-ASA + cortic
18	17	M	Smoker	22,53	Moderate	2	Severe	5-ASA + cortic
19	62	M	4 cig/day	25,27	Moderate	3	Mild	5-ASA + cortic
20	30	M	No	22,86	Moderate	2	Severe	Cortic + AZA
21	42	W	No	27,34	Mild	2	Severe	5-ASA + cortic
22	73	M	20 cig/day	26,95	Moderate	2	Mild	5-ASA + cortic
23	44	M	Ex-smoker	23,98	Moderate	2	Severe	5-ASA + cortic + IMM
24	62	W	No	24,22	Mild	2	Moderate	5-ASA + cortic

1 Data from each patient were collected retrospectively from medical records including age, sex, smoke history, Body Index Mass (BMI), endoscopic (Mayo clinic score) and clinical score (Truelove and Witts score: mild, moderate and severe) at onset, histological features and treatments received since initial diagnostic (5-aminosalicilates, 5-ASA; corticosteroids; and/or the immunomodulators, IMM, Cyclosporine A and/or Azathioprine).

### Immunohistochemistry

We analyzed the distribution of CB_1_ and CB_2_ receptors, FAAH, MAGL, DAGLα, DAGLβ, and NAPE-PLD in the normal colonic tissue and in the acute and quiescent UC mucosa by immunohistochemistry, following methods previously described[34], [35]. Tissue blocks were fixed in 4% (w/v) buffered formaldehyde and embedded in paraffin. Blocks were cut into longitudinal 5-µm-thick sections using a Microm HM325 microtome (MICROM, Walldorf, Germany). Sections were mounted on glass slides with the positively charged surface (DAKO Real, ref. S2024, Glostrup, Germany) and air-dried. After the sections were dewaxed, antigen retrieval was achieved through incubating in H_2_Od containing 50 mM sodium citrate (pH 9) for 15 minutes at 80°C, followed by washes in 0,1M phosphate-buffered saline (PBS; pH 7.4). Then incubation in 3% hydrogen peroxide (H_2_O_2_) for 20 minutes was achieved to inactivate the endogenous peroxidase. Later, sections were blocked in 10% donkey serum in PBS and 0.1% NaN_3_ for 1 hour, and incubated overnight at room temperature with the following antibodies34: anti-CB1 receptor (diluted 1∶100; ABR, cat. no. PA1-745, lot. no. 424-121); anti-CB2 receptor (diluted 1∶100; ABR, cat. no. PA1-746A, lot. no. 452-114); anti-FAAH (diluted 1∶100; Cayman, cat. no. 101600, lot. no. 157878); anti-MAGL (diluted 1∶100; Cayman, cat. no. 100035, lot. no. 163084); anti-NAPE-PLD, diluted 1∶100; anti-DAGLα, diluted 1∶50; and anti-DAGLβ, diluted 1∶50 (supporting information S1). Then, the sections were incubated in a biotinylated donkey anti-rabbit immunoglobulin (Amersham) diluted 1∶500 for 1 hour, and incubated in ExtrAvidin peroxidase (Sigma) diluted 1∶2000 for 1 hour. We revealed immunolabeling with 0.05% diaminobenzidine (DAB; Sigma), 0.05% nickel ammonium sulphate, and 0.03% H_2_O_2_ in PBS. Al steps were carried out in PBS with gently agitation at room temperature. Sections were dehydrated in ethanol, cleared in xylene, and coverslipped with Eukitt mounting medium (Kindler GmBH and Co., Freiburg, Germany).

Digital high-resolution microphotographs were taken under the same conditions of light and brightness/contrast by an Olympus BX41 microscope equipped with an Olympus DP70 digital camera (Olympus Europa GmbH, Hamburg, Germany). Digital images were mounted and labelled using Adobe PageMaker (San Jose, CA, USA).

### Western blotting

We collected prospectively 8 rectal samples of control patients underwent colonic resection biopsies, processed as previously described [34], [35], to evaluated the presence of CB_1_ and CB_2_ receptors, FAAH, MAGL, DAGLα, DAGLβ and NAPE-PLD by Western blotting. Samples from were immediately snap frozen in liquid nitrogen and stored at −80°C until use. Membrane extracts of colon tissue were prepared in HEPES 50 mM (pH 8)-sucrose 0.32 M buffer by using a homogenizer. The homogenate was centrifuged at 800 xg for 10 minutes at 4°C and the supernatant was centrifuged at 40000 xg for 30 minutes. The pellet was suspended in HEPES 50 mM buffer and potterized using a homogenizer.

For immunoblotting, equivalent amounts of membrane proteins (20 µg) were separated by 10% sodium dodecyl sulphate-polyacrylamide gel electrophoresis (SDS-PAGE), electroblotted onto nitrocellulose membranes, and controlled by Ponceau red staining. Blots were preincubated with a blocking buffer containing PBS, 0.1% Tween 20 and 2% albumin fraction V from bovine serum (Merck, Whitehouse Station, NJ, USA) for 1 h at room temperature. For protein detection, each blotted membrane lane was incubated separately with the specific CB_1_ (1∶250), CB_2_ (1∶300), FAAH (1∶200), MAGL (1∶200), DAGLα(1∶200), DAGLβ (1∶200) and NAPE-PLD (1∶100) antibodies, diluted in the blocking buffer, overnight at 4°C. After extensive washing in PBS containing 1% Tween 20 (PBS-T), a peroxydase-conjugated goat anti-rabbit antibody (Promega, Madison, WI, USA) was added (1∶10000) for 1 h at room temperature. Biotinylated marker proteins with defined molecular weights were used for molecular weight determination in Western blots (ECL™ Western Blotting Molecular Weight Markers, Amersham/GE Healthcare, Buckinghamshire, UK). Membranes were subjected to repeated washing in PBS-T and the specific protein bands were visualized using the enhanced chemiluminiscence technique (ECL, Amersham) and Auto-Biochemi ™ Imaging System (LTF Labortechnik GmbH, Wasserburg/Bodensee, Germany). Western Blots showed that each primary antibody detects a protein of the expected molecular size.

As controls, we incubated blotted membrane lanes with the primary antibody preadsorbed with the immunizing peptides (Table 2): CB_1_ and CB_2_ (both at 20 µg/ml; kindly donated by Dr. K. Mackie), FAAH (10 µg/ml; Cayman, lot. no. 301600), MAGL (5 µg/ml; Cayman, lot. no. 300014), DAGLα, DAGLβ and NAPE-PLD (25 µg/ml, 100 µg/ml and 25 µg/ml respectively; JPT, Berlin, Germany). We did not detect staining under these conditions.

**Table 2:** Immunizing peptides used in this study.

Proteins	Peptides sequences	GenPept accession number
**CB_1_**	MKSILDGLADTTFRTITTDLLYVGSNDIQYEDIKGDMASKLGYFPQKFPLTSFRGSPFQEKMTAGDNSPLVPAGDTT	NP_036916.1
**CB_2_**	MAGCRELELTNGSNGGLEFNPMKEYMILSDAQ	NP_065418.2
**NAPE-PLD**	MDENSCDKAFEET	NP_955413.1
**DAGLα**	CGASPTKQDDLVISAR	NP_001005886.1
**DAGLβ**	SSDSPLDSPTKYPTLC	NP_001100590.1
**FAAH**	CLRFMREVEQLMTPQKQPS	NP_077046.1
**MAGL**	SSPRRTPQNVPYQDL	Q8R431.1

### Quantification of mucosa immunostaining

One immunostaining batch contained 70 tissue sections of all experimental groups, thus slices corresponding to the three experimental groups were stained simultaneously. For each primary antibody and for each subject, 2–3 different batches were run. On each tissue section we focussed on epithelium and lamina propria of the mucosa. For epithelium, we carried out a densitometrical quantification for each component of the ECS. For lamina propria, we evaluated the type and the number of immunostained immune cells for each 100 cells observed by hematoxylin-eosin (H&E) staining. In addition, ECS quantification was segregated depending on UC severity scored to mild, moderate and severe (Truelove and Witts score), and by the treatment received (5-ASA, corticosteroids, and/or the immunomodulators).

Digital high-resolution microphotographs were taken with the 10× objective of an Olympus BX41 microscope under the same conditions of light and brightness/contrast. Quantification of immunostaining was carried out by measuring densitometry of the selected areas using the analysis software ImageJ 1,38× (Rasband,W.S., ImageJ, National Institute of Health, Bethesda, Maryland, USA).

### Statistical analysis

Data were analyzed using SPSS 15.0 software (Statistical Package for the Social Sciences Inc., Chicago, Illinois, USA). Results are expressed as mean±SEM. Differences between groups were evaluated using U Mann Witney and Wilcoxon tests for non parametric observations. A *P* value of *P*<0.05 was considered statistically significant.

## Results

### Presence of the endocannabinoid system in the normal human colonic tissue: Western blot analysis

**Figure 1:**
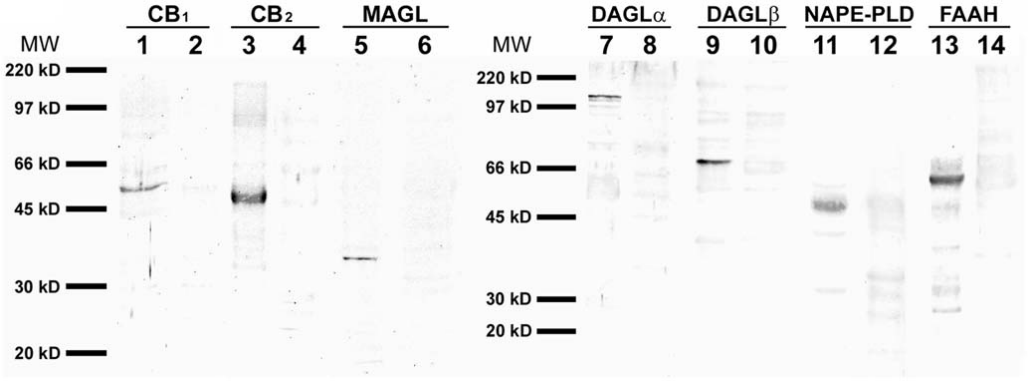
Western blots of membrane extracts from human colonic tissue. They showed prominent immunoreactive bands of the expected size for the ECS proteins. See text. Positions of molecular markers (MW) are indicated at the left.

Western blot analysis of membrane proteins from normal human colon tissue revealed the presence of all ECS proteins studied. They appeared as prominent bands of 53 kD for CB1, (fig. 1, lane 1), 50 kD for CB_2_ (fig. 1, lane 3), 35 kD for MAGL (fig. 1, lane 5), 120 and 73 kD for DAGLα and DAGLβ respectively (fig. 1, lanes 7 and 9), and 46 and 63 kD for NAPE-PLD and FAAH respectively (fig. 1, lanes 11 and 13). In each case, the immunoreactive bands were abolished after adsorption with the immunizing peptides (fig. 1, lanes 2, 4, 6, 8, 10, 12, 14).

### Immunohistochemical distribution of the endocannabinoid system in the normal human colonic tissue

**Figure 2:**
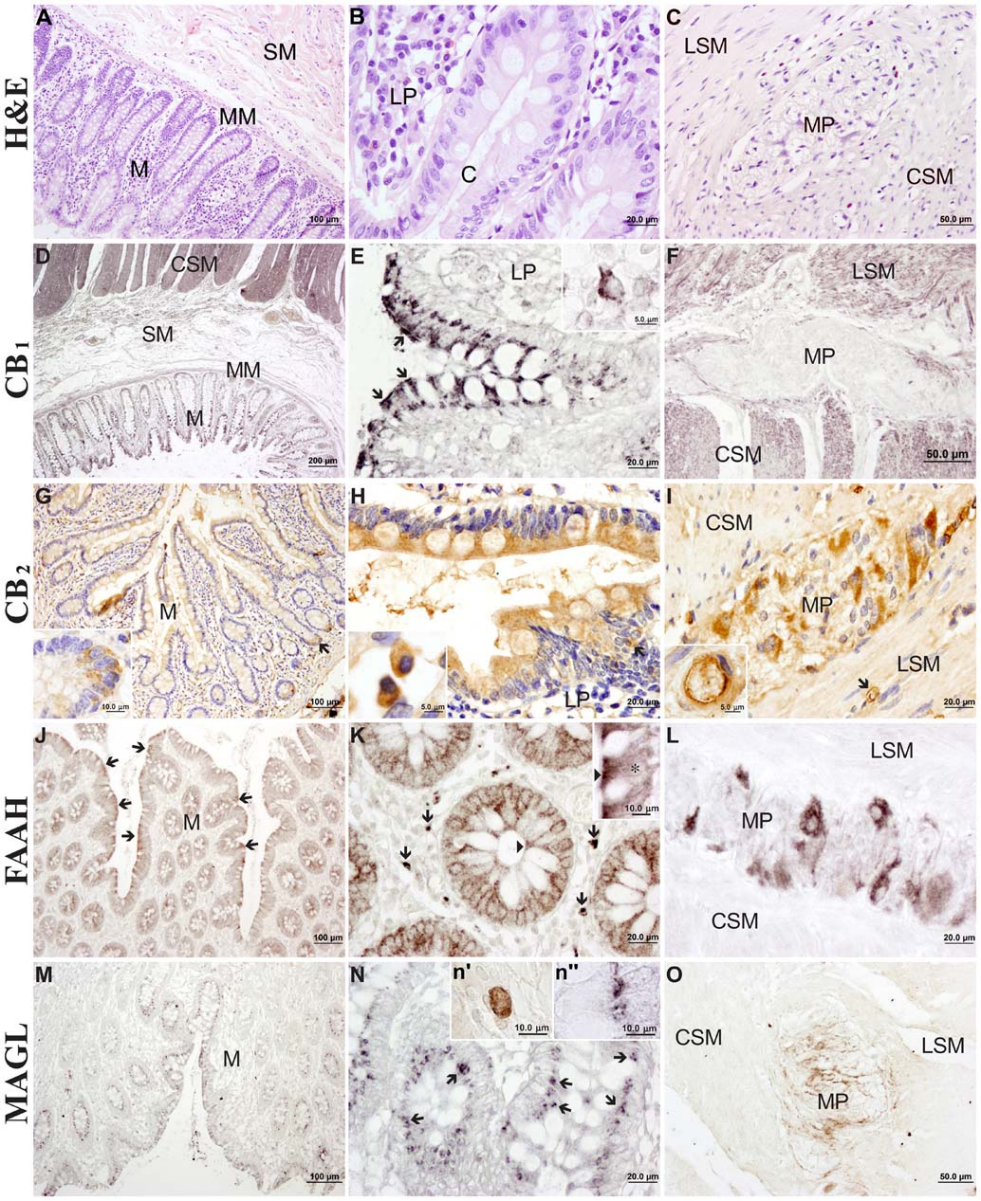
Immunohistochemistry for CB_1_ and CB_2_ receptors, FAAH and MAGL in human colonic tissue. Morphology of normal human colon, stained with H&E (A–C). General views of transmural sections through the colon (A, D, G, J, M). High-magnification photomicrographs of the colonic epithelium and lamina propria (B, E, H, K, N), smooth muscle and myenteric plexus (C, F, I, L, O). *Abbreviations*: C, crypt; CSM, circular smooth muscle; LP, lamina propria; LSM, longitudinal smooth muscle; M, mucosa; MM, muscularis mucosae, MP, myenteric plexus; SM, submucosa.

Results for the immunohistochemical distribution were summarized in a rating scale (Table 3). Intense CB_1_ immunoreactivity is showed in the epithelial cells of the crypts (C), being prominent in the absorptive cells, mainly on the apical surface facing the lumen (fig. 2D, E, arrows). We observe CB_1_ immunoreactivity in some plasma cells of the lamina propria (LP; fig. 2E, inset). A low/moderate staining was detected in the muscularis mucosae (MM), including the smooth muscle of the blood vessels, but intensely staining characterized inner circular (CSM) and outer longitudinal (LSM) smooth muscle layers (fig. 2D, F). Of note, the varicose aspect of CB_1_ immunoreactivity on the muscle cells that probably consist of nerve terminals (fig. 2F, inset). We observed faintly immunostaining in the parasympathetic nervous cells of both Meisnner's and myenteric plexi (MP), except of some scattered fibers (fig. 2F). Some CB_1_+ connective cells were also detected in the serosa layer.

**Table 3:** Immunoreactivity of endocannabinoid system in normal colonic tissue (n = 22)^1^.

	Epithelium Glands	Lamina propria	Smooth muscle	Myenteric plexus
CB_1_	+++	−	++	−
CB_2_	+++	+	+	++++
FAAH	++	++	−	++++
MAGL	++	++	−	++
NAPE-PLD	+++	++	+++	−
DAGLα	+++	++	++++	++
DAGLβ	++++	++	+++	++++

1 Gray-scale values measured in single epithelium, lamina propria, muscular layers and plexi are represented on an arbitrary rating scale of the immunoreactivity of each structure. Symbols are as follows: very high (++++), high (+++), low (++), very low (+) and without immunoreactivity (−).

CB2 immunoreactivity was detected in the colonic epithelium of both absorptive and goblet cells (fig. 2H). Of note, a stronger CB_2_ immunoreactivity in the Paneth cells, at the bottom of the crypts, than in the remaining colonic epithelium (fig. 2G inset). A number of subepithelial CB_2_+ plasma cells and probably some macrophages were detected in the lamina propria (fig. 2H arrow, inset). We also observed weak CB_2_ immunoreactivity in the muscularis mucosae and muscularis externa whereas intense staining was located in the endothelial cells of the blood vessels (fig. 2I arrow, inset). Numerous CB_2_+ ganglion cells and fibers were evident in the myenteric plexus (fig. 2I) and the submucosal plexi.

FAAH immunostaining disposed in the epithelial cells, being prominent in the apical one third and perinuclear portions of the absorptive cells (fig. 2J, K inset, asterisk). The brush border of the microvilli was nearly absent of staining (fig. 2K inset, arrowheads). We detected few scattered FAAH+ immune plasma cells in the lamina propria. No staining was observed neither in the muscularis mucosae, muscularis externa or serosa, whereas intense staining was observed in some ganglion cells and fibers of the myenteric plexus (fig. 2L).

MAGL immunoreactivity was located in the central portion of the epithelial cells, thus, apical to the nucleus of the absorptive cells and basal to the mucus droplets of the goblet cells (fig. 2M, N, inset n″). A number of immunoreactive polymorphonuclear cells was distinguished in the lamina propria (fig. 2N, inset n′). No staining was detected in both muscularis mucosae and externa. The myenteric plexus was characterized by a meshwork of MAGL+ fibers that disposed surrounding unstained parasympathetic nervous cells (fig. 2O).

**Figure 3:**
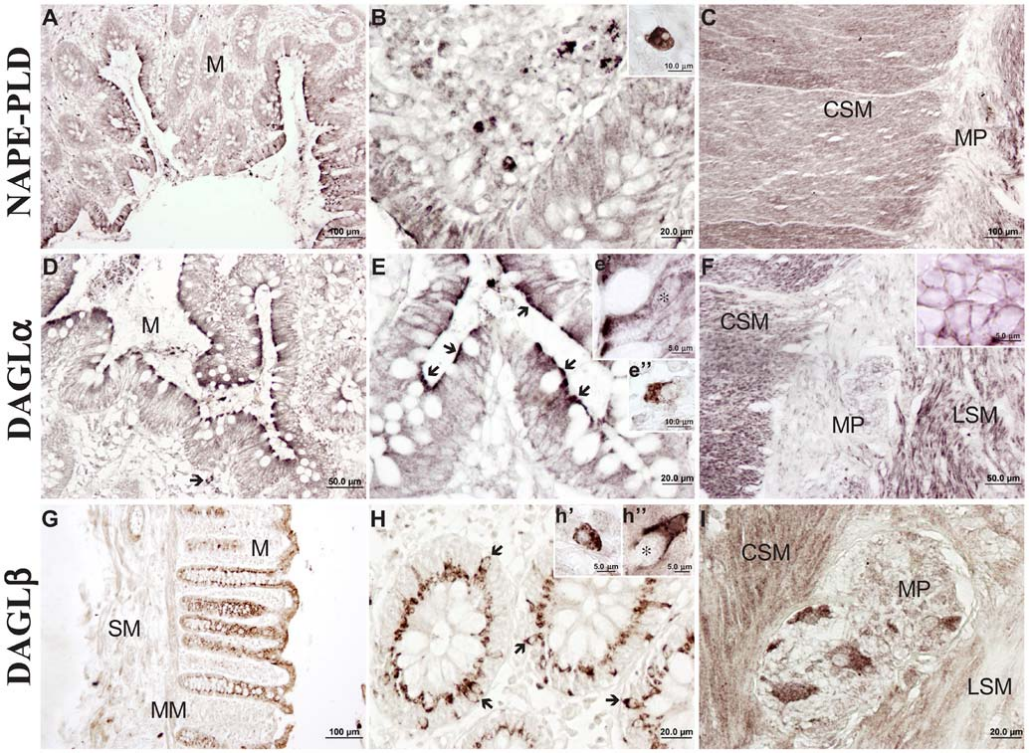
Immunohistochemistry for NAPE-PLD, DAGLα and DAGLβ in human colonic tissue. General views of transmural sections through the colon (A, D, G). High-magnification photomicrographs of the colonic epithelium and lamina propria (B, E, H), smooth muscle and myenteric plexus (C, F, I).

Strong NAPE-PLD immunoreactivity in the apical surface of the epithelial border of the crypts (fig. 3A) and numerous positive plasma cells was observed (fig. 3B, inset). Intense NAPE-PLD immunostaining characterized both layers of muscularis externa (fig. 3C). Numerous immunoreactive fibers, but not cell bodies, disposed in the myenteric plexus (fig. 3C).

We observed a similar DAGLα staining pattern in the colonic tissue to that of CB_1_ and NAPE-PLD proteins (fig. 3D). An intense immunoreactivity characterized the apical surface of epithelial border facing to lumen (arrows in fig. 3E, inset e′). We observed some DAGLα+ plasma cells in the lamina propria (fig. 3E, inset e″). Muscularis mucosae and externa showed an intense DAGLα immunoreactivity (fig. 3F) in a similar granular aspect to that of CB_1_ immunoreactivity. Numerous DAGLα+ fibers disposed surrounding unstained ganglion cells in the myenteric plexus (fig. 3F, inset).

Intense DAGLβ expression was mainly located surrounding the nucleus of the epithelial cells (fig. 3G, H, inset h″). A number of scattered plasma cells also showed intense DAGLβ staining (fig. 3H, inset h′). Muscularis mucosae appeared positive, but strongly DAGLβ expression was evident in both layers of the muscularis externa, mainly in the inner one (fig. 3I). The myenteric plexus was characterized by strongly DAGLβ+ ganglion cells and a dense fibre network (fig. 3I).

### Densitometrical quantification of ECS immunoreactivity in the colonic epithelium

**Figure 4:**
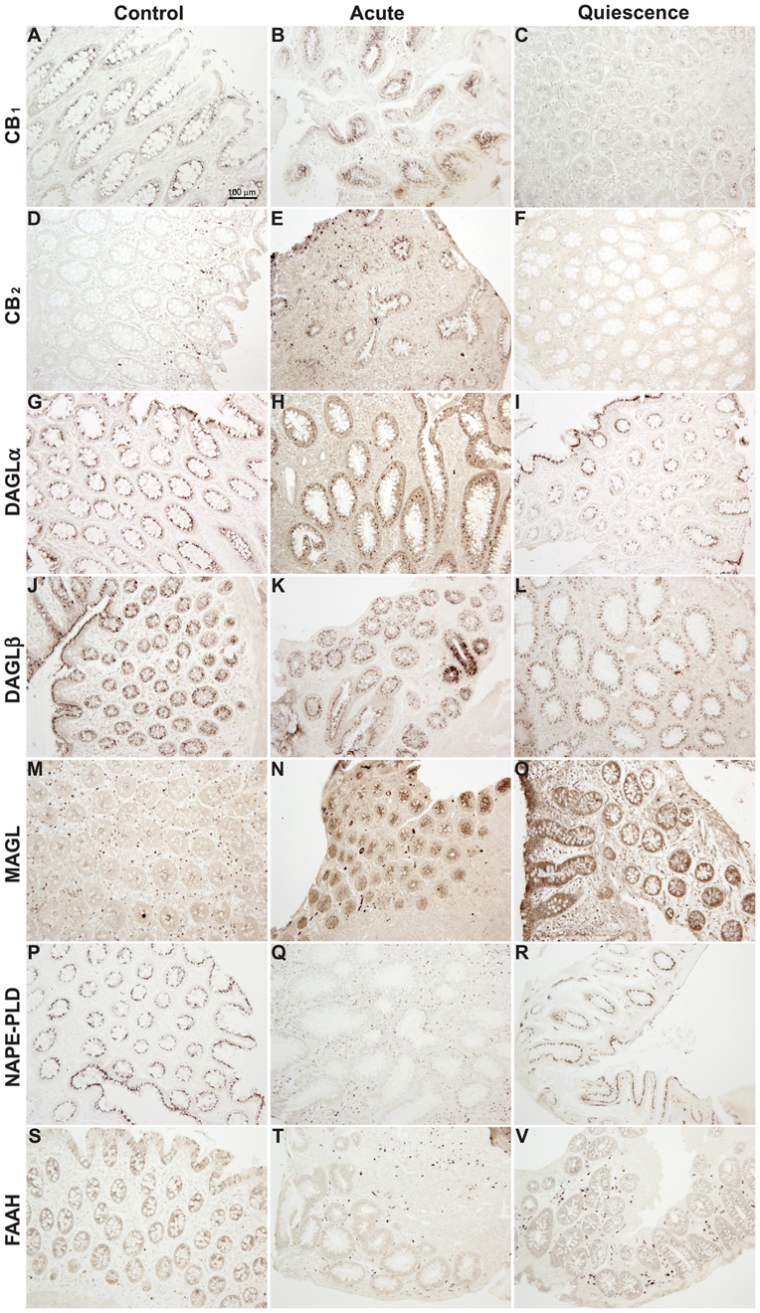
Immunohistochemistry in human healthy (control), acute UC and quiescent UC colonic tissue. Representative microphotographs of CB_1_ receptor (A–C), CB_2_ receptor (D–F), DAGLα (G–I), DAGLβ (J–L), MAGL (M–O), NAPE-PLD (P–R), FAAH (S–V) were shown.

Microphotographs showing qualitative differences of the immunoreactivity for each ECS component in the epithelium of control, acute and quiescent groups are shown in figure 4.

**Figure 5:**
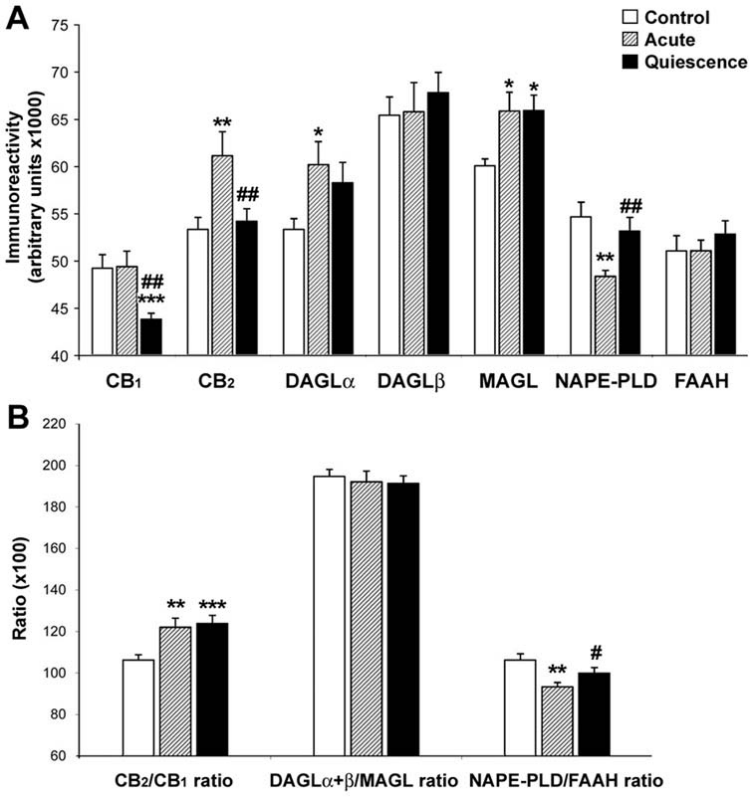
Quantification of ECS component immunoreactivity in the colonic epithelium. A: Untreated acute UC at disease onset showed increases in CB_2_, DAGLα and MAGL immunoreactivity, and decreases in NAPE-PLD immunostaining. After achieving remission (quiescence), CB_1_ and CB_2_ receptor immunoreactivity dropped, MAGL immunostaining maintained the same levels than acute group and NAPE-PLD immunoreactivity reverted to control levels. B: CB_2_/CB_1_ ratio increased in both groups. However, CB_2_ immunoreactivity increased in acute patients, while in quiescent patients there was a decrease of CB_1_ receptor and a reverted restoration of CB_2_ level. NAPE-PLD/FAAH ratio dropped in acute group, but rose to control levels in quiescent one. Histograms represent the mean±SEM. U Mann Witney and Wilcoxon tests: *P<0.05, **P<0.01 and ***P<0.001 versus control group; #*P*<0.05 and ##*P*<0.01 versus acute group. N = 22, 24 and 24 for control, acute and quiescent groups respectively.

Quantification of epithelial immunoreactivity for ECS components is shown in figure 5. CB_1_ expression was maintained in acute group [49.18±1.44 vs 49. 37±1.62 (×10^3^)] but, in quiescent group, was lower than in control one [44.75±1.22 vs 49.18±1.44 (×10^3^); p<0.001], as well as when was compared with the acute one [44.75±1.22 vs 49.37±1.62 (×10^3^); p<0.01], suggesting that CB_1_ receptor may be downregulated by the treatment. We detected an increase of CB_2_ expression in acute group comparing with the control one [61.09±2.54 vs 53.30±1.27 (×10^3^); p<0.01]. In contrast, increased CB_2_ expression was reversed in quiescent group [61.09±2.54 vs 55.15±1.69 (×10^3^); p<0.01]. These data may indicate an overexpression of CB_2_ receptor during the acute inflammation but, once controlled by the treatment, restored to basal levels. However, the increased ratio in acute samples was due to an increase of CB_2_ receptors [1.22±0.04 vs 1.06±0.02; p<0.01] whereas in quiescent samples it was derived from a downregulation of CB_1_ receptors [1.23±0.039 vs 1.06±0.02; p<0.001].

Enzymes of 2-AG pathway were overexpressed in UC patients; in acute and quiescent groups in comparison with control one. DAGLα and MAGL were significantly increased in acute group regarding control one [62.79±3.71 vs 53.79±1.29 (×10^3^) for DAGLα; 65.81±1.99 vs 60.81±0.94 (×10^3^) for MAGL; p<0.05]. However, DAGLα increase in quiescent group did not reach statistical significance when compared with control group [58.22±2.16 vs 53.79±1.29 (×10^3^); p = 0.06]. In contrast, MAGL increase was statistically maintained between quiescent and control groups [65.85±1.64 vs 60.81±0.94 (×10^3^); p<0.01]. These data suggest an increase of 2-AG turnover during the inflammation and a decrease after achieving remission. No statistical differences in DAGLβ expression were observed between control, acute and quiescent groups. However, the DAGLα+β/MAGL ratio, an estimation of the balance of 2-AG levels, did not change either in acute or quiescent patients.

NAPE-PLD immunoreactivity was significantly decreased in acute group in comparison with control one [49.46±1.38 vs 54.63±1.56 (×10^3^); p<0.01]. NAPE-PLD expression in quiescent group recovered to control levels [53.11±1.46 vs 54.63±1.56 (×10^3^)], being this increase statistically significant when compared with acute group [53.11±1.46 vs 49.46±1.38 (×10^3^); p<0.01]. No statistical differences in FAAH expression were found between control, acute and quiescent groups. The NAPE-PLD/FAAH ratio, an estimation of AEA balance, decreased in acute group in comparison with control group (0.93±0.02 vs 1.06±0.03; p<0.01), and increased to control levels in quiescent group when was compared with acute group (0.99±0.02 vs 0.93±0.02; p<0.05). These data suggest a dysregulation of the AEA balance in the acute inflammatory process that recovers to control level after treatment.

### Percentage of the ECS immunoreactive cells in the lamina propria

**Figure 6:**
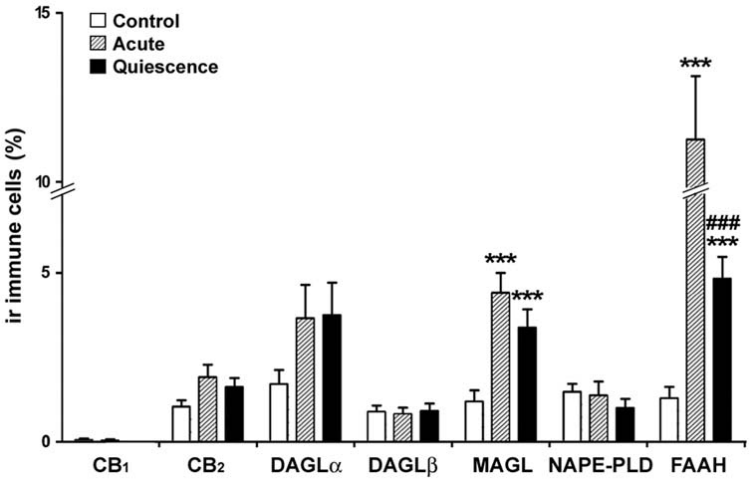
Percentage of immunoreactive immune cells for ECS components in the lamina propria. Untreated acute UC at disease onset is associated with high number of FAAH+ and MAGL+ immune cells that was significantly diminished after treatment only in FAAH immunoreactivity. Histograms represent the mean±SEM. U Mann Witney and Wilcoxon tests: ***P<0.001 versus control group; ###*P*<0.001 versus acute group. N = 22, 24 and 24 for control, acute and quiescent groups respectively.

We found pronounced changes in the number of FAAH+ and MAGL+ cells, but not to the remaining ECS components (fig. 6). FAAH+ cell number rose in acute group compared with control one (11.2%±1.9% vs 1.29%±0.3%; p<0.001). Besides, a decrease in the number of FAAH+ cells was evidenced in quiescent group compared with acute group (4.8%±0.6% vs 11.2%±1.9%; p<0.001) but was still notably higher than in controls (p<0.001).

We found higher percentage of MAGL+ cells in acute and quiescent groups than in controls (4.4%±0.5% vs 1.2%±0.3%; 3.4%±0.5% vs 1.2%±0.3%; p<0.001).

### Quantification of epithelial ECS immunoreactivity depending on the severity of the UC disease

**Figure 7:**
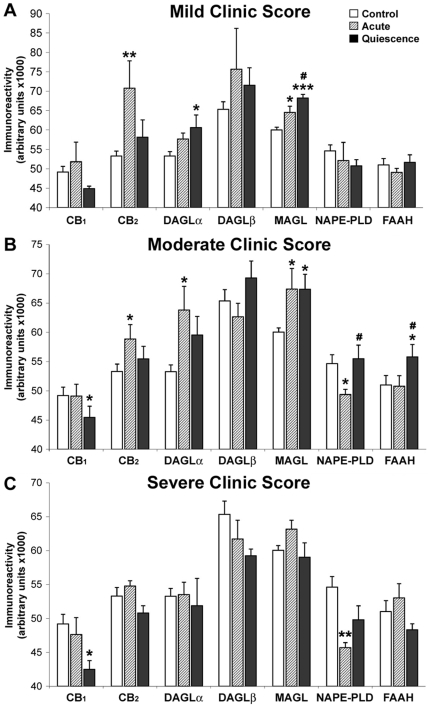
Quantification of epithelial ECS immunoreactivity depending on the UC severity score. Main changes were observed mainly in mild and moderate acute UC. Histograms represent the mean±SEM. U Mann Witney and Wilcoxon tests: *P<0.05, **P<0.01 and ***P<0.001 versus control group; #*P*<0.05 versus acute group. N = 6, 13 and 5 for mild, moderate and severe groups respectively.

We compared ECS in acute group depending on the severity of the disease and after remission (quiescent group) vs control tissue (fig. 7). CB_1_ expression did not change in acute samples at any clinic score. In quiescent samples, CB_1_ expression dropped significantly in moderate UC flare patients [45.46±1.91 vs 49.18±1.44 (×10^3^); p<0.05] or severe [42.48±1.32 vs 49.18±1.44 (×10^3^); p<0.05], in comparison with controls (Fig. 6). In mild UC, the decrease did not reach the significance between quiescent and control groups [44.89±0.64 vs 49.18±1.44 (×10^3^); p = 0.06].

Intense CB_2_ immunoreactivity in acute group was evidenced in mild [70.801±7.042 vs 53.301±1.278 (×10^3^); p<0.01] and moderate colitis [58.86±2.46 vs 53.30±1.27 (×10^3^); p<0.05], in comparison with controls but not in the severe cases. There was no change in CB_2_ immunoreactivity between quiescent and control samples.

We only found a rise of DAGLα expression in acute moderate colitis compared with control groups [61.21±3.20 vs 53.28±1.16 (×10^3^); p<0.05]. In mild colitis patients, higher levels of DAGLα were also observed in quiescent samples compared with controls [55.67±2.93 vs 53.28±1.16 (×10^3^); p<0.05]. No differences in DAGLβ expression were observed among the three clinic scores. Regarding NAPE-PLD, no differences were found in mild colitis among the three groups, but when we compared acute group with controls as the severity raises the expression drops. Differences were significant in moderate [49.37±0.88 vs 54.63±1.56 (×10^3^); p<0.05] and severe colitis [45.70±0.74 vs 54.63±1.56 (×10^3^); p<0.01]. NAPE-PLD immunoreactivity rose to control values in quiescent stage of moderate colitis compared with acute group [52.34±6.68 vs 49.37±3.18 (×10^3^); p<0.05].

Higher levels of FAAH immunoreactivity were measured in quiescent samples of moderate UC patients compared with acute [55.78±2.15 vs 50.79±1.80 (×10^3^); p<0.05] and control samples [55.78±2.15 vs 51.01±1.63 (×10^3^); p<0.05]. No changes of FAAH expression were detected in acute or quiescent groups from mild and severe clinic score patients. In mild and moderate colitis, we evidenced higher expression of MAGL in acute [64.57±1.60 vs 60.03±0.72 (×10^3^) in mild; 67.41±3.49 vs 60.03±0.72 (×10^3^) in moderate; p<0.05] and quiescent [68.25±0.96 vs 60.03±0.72 (×10^3^) in mild; 67.36±2.54 vs 60.03±0.72 (×10^3^) in moderate; p<0.001 and p<0.05 respectively] stages compared with controls. In mild UC these levels were even higher in quiescent stage than in acute one (p<0.05). No differences were seen in severe colitis among the three groups.

### Quantification of epithelial ECS immunoreactivity depending on treatment

**Figure 8:**
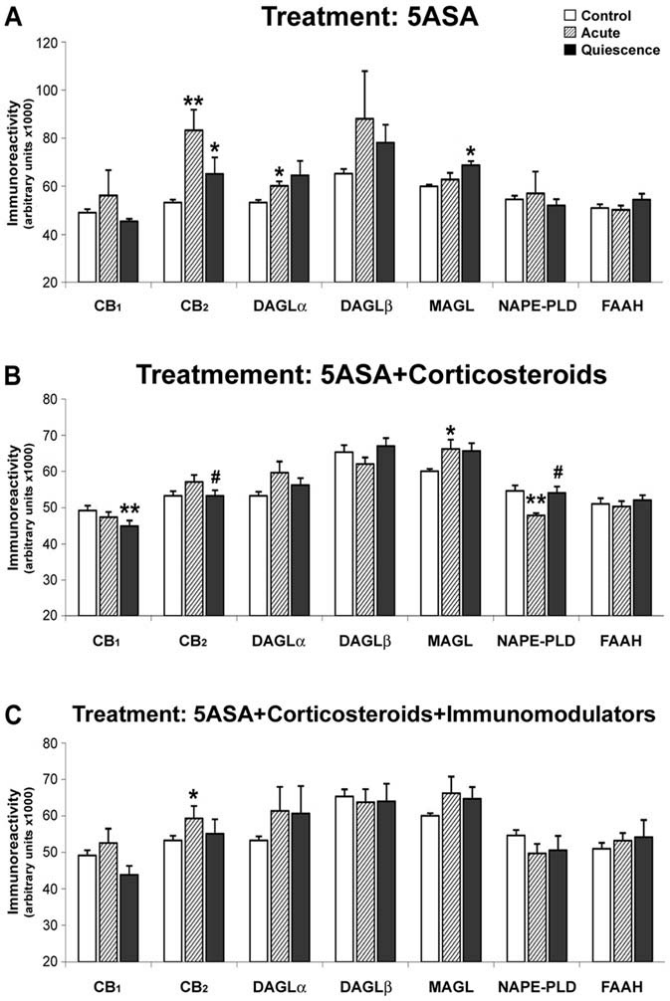
Quantification of epithelial ECS immunoreactivity depending on the treatment received. As a relevant finding, treatment is associated with changes in the expression of cannabinoid receptors and EC-production and -degradation enzymes, suggesting that these proteins can be considered as biomarkers of active disease/response to treatment. Histograms represent the mean±SEM. U Mann Witney and Wilcoxon tests: *P<0.05 and **P<0.01 versus control group; #*P*<0.05 versus acute group. N = 3, 15 and 6 for 5-ASA, 5-ASA+corticosteroids and 5-ASA+corticosteroids+immunomodulators respectively.

We analyzed ECS immunoreactivity in quiescent samples depending on the treatment received: 5-ASA (3 cases), 5-ASA and corticosteroids (15 cases), or 5-ASA, corticosteroids and immunomodulators (6 cases) (fig. 8). Regarding CB_1_ levels, there was a decrease in patients treated with 5-ASA+corticosteroids [49.18±1.44 vs 44.91±1.58 (×10^3^); p<0.01] but not with other treatments. By contrast, CB_2_ and MAGL expression increased in 5-ASA-treated patients but not after the remaining treatments [57.20±1.87 vs 53.29±1.52 (×10^3^) for CB_2_; 68.78±1.78 vs 60.03±0.72 (×10^3^) for MAGL; p<0.05]. DAGLα, DAGLβ, NAPE-PLD and FAAH expression were not altered by the treatment.

## Discussion

Our data are consistent with previous studies on the expression of CB_1_ and CB_2_ receptors in human and rodent colon.[20], [21], [36], [37] A novelty of our study is the finding of CB_1_ staining in the goblet cells. Interestingly, the previous human study[20] did not report CB_1_ staining in the goblet cells probably as a result of mucus-blocking antibody binding. Casu and collaborators[21] described non-specific labelling in the murine colonic epithelial cells of the large intestine because it persisted in preabsorption and omission controls. In contrast, we observed faintly CB_1_ immunoreactivity in the submucosal and myenteric ganglion plexi, with the exception of some fibers. The well-described presynaptic localization of CB_1_ receptor contrasts with the presence of this receptor into submucosal ganglion cell bodies, as was described in the human and mouse colon.[20], [21], [37] Our results revealed similar CB_2_ expression in the mucosal epithelial cells from normal patient samples in a previous human colonic study that, using different CB_2_ antibodies, supports our immunohistochemical data.[20] Of note, we observed strong CB_2_ expression in the Paneth cells at the bottom of the crypts. CB_2_+ subepithelial plasma cells and macrophages in the lamina propria was described previously by Wright and collaborators.[20], [22] A novelty data was the finding of CB_2_ staining in the submucosal and myenteric plexi of the normal human colonic tissue. Recently, CB_2_ expression was observed in the enteric nervous system in rodent and human ileum[19], [22], and in the rat ileum containing longitudinal muscle and myenteric plexus.[38] Taking together these results point to a differential role of cannabinoid CB_1_ and CB_2_ receptors in human colonic tissue. CB_1_ could be modulating colonic neuronal input and secretion while CB_2_ may participate in colonic immunomodulation.

Other important novelty is the presence of the two endocannabinoid-degradating enzymes (FAAH and MAGL) in the epithelial cells of human colonic tissue. We have clearly detected FAAH expression in plasma cells of the lamina propria and in ganglion cells of the enteric nervous system. These results are related to the fact that FAAH blockers like URB597 reduce significantly the inflammation in the mouse colon[28], and selective FAAH inhibitors like AA-5-HT inhibited intestinal motility.[39] MAGL localization into epithelial cells is in agreement with the presence of MAGL activity in the soluble and membrane cellular fractions.[40] Of note the immunoreactive polymorphonuclear cells in the lamina propria, a fact that has not been observed previously. In contrast to Duncan and collaborators[40], we did not observed MAGL immunoreactivity in the human smooth muscle and mucosal layers, but we detected MAGL expression in fibers of the enteric nervous system.

We have reported the first analysis of the presence of DAGLα, DAGLβ and NAPE-PLD in the human colonic tissue. Although 2-AG is considered a full cannabinoid receptor agonist, it is also an intermediate in triacyl/diacylglycerol metabolism as well as a prominent molecule linking the cannabinoid signalling with lysophospholipids and diacilglycerol-PKC signalling system. However, although we cannot strictly consider both DAGLα and DAGLβ as pure endocannabinoid-synthesizing enzymes, we will focus on their potential role in the endocannabinoid system.

On the other hand, NAPE-PLD is another recently characterized cannabinoid biosynthesis enzyme that mediates the release of N-acyl ethanolamides (including AEA) from a phospholipid precursor (N-acyl-phosphatidylethanolamide, NAPE).[15], [41] Our results are compatible with an active synthesis of ECs, i.e. AEA and 2-AG, in healthy human colonic tissue.

There are higher levels of cannabinoid CB_2_ receptors (but not CB_1_ receptors) in the mucosa epithelium of UC, mainly in mild and moderate-scored patients. These data suggest a dysregulated AEA tone in the colon of these patients, in agreement with previous findings.[20], [25] However, we observed low NAPE-PLD expression, mainly in moderate and severe-scored pancolitis patients, and no changes in the AEA-degrading enzyme FAAH, suggesting a decrease of AEA levels, as deduced by the NAPE-PLD/FAAH ratio, while D-Argenio et al. found high AEA levels in biopsy samples of colons from untreated UC patients.[25] This discrepancy may be explained by the fact that NAPE-PLD is not the only source for AEA, as others enzymes are also capable of generating AEA from NAPE, such as α/β hydrolase 4, lyso-PLD, lyso-PLC, and phosphatases such as PTPN22.[42]–[44] Thus, although we detect a dysregulated AEA tone, the whole changes of AEA-related enzymes could lead to an increased level of this EC.

Regarding 2-AG, we observed an increase of DAGLα and MAGL expression in the colonic epithelium of acute UC patients, suggesting an increase of 2-AG turnover during the inflammation, but not a dysbalance of 2-AG levels, as suggest the DAGL/MAGL ratio. The maintenance of DAGL/MAGL ratio is in agreement with the absence of 2-AG variations observed in the mucosa of TNBS-treated rats, DNBS-treated mice and UC patients.[25] The high DAGLα and DAGLβ expression detected in the human colonic epithelium may be partially related with the high 2-AG levels described in colonic mucosa of untreated rats, in contrast to that of control patients.[25]

Interestingly, severe clinic score patients showed no significant increase in CB_2_ receptors, and this fact correlates with a lack of increased 2-AG turnover (no increases of synthesizing- and degrading enzymes), thus suggesting a diminished ECS response to the inflammatory insult. In light of these findings, we could speculate that ECS-related drugs potentiating ECs turnover could be useful in managing the disease in this subpopulation of patients.

Regarding the cannabinoid receptors in treated UC, the acute CB_2_ increase in UC patients is reverted in the chronic state, irrespective of the treatment. This fact suggests a putative role of CB_2_ receptor in mediating acute inflammatory response. In addition, the treatments, mainly the 5-ASA+corticosteroids one, lead to a chronic down-regulation of CB_1_ receptor (not displayed acutely), probably reflecting a diminished colonic functionality in the chronic state of the disease, since CB_1_ receptor have been implicated in colonic motility and secretion.[27], [39] Thus, cannabinoid CB_1_ receptor could be a biological marker of UC progression. Interestingly, while the high MAGL expression is maintained in quiescent patients, NAPE-PLD expression recovered to control levels, suggesting a partial recovery of the ECS dysregulation after treatments.

In summary, these data indicate that endocannabinoid signaling pathway is altered in UC, acting probably through cannabinoid CB_2_ receptor as a counteregulatory system aimed to reduce colitis-associated inflammation. In addition, the changes observed in the remaining ECS components, both acutely and after treatment, suggest that drugs acting at the ECS could be potential therapeutic approaches that need to be explored in more depth, for the treatment of inflammatory bowel diseases.

## Supporting Information

Supporting Information S1 Generation of NAPE-PLD-, DAGLα-, DAGLβ-specific antibodies. We have generated polyclonal rabbit antibodies against proteins of the cannabinoid machinery. Immunizing peptides were 1) a 13-amino-acid (aa) peptide comprising part of both the C-terminal and the N-terminal regions of NAPE-PLD (MDENSCDKAFEET); 2) a 16-aa peptide from the C-terminal region of DAGL alpha (CGASPTKQDDLVISAR); 3) a 16-aa peptide from an internal sequence of DAGL beta (SSDSPLDSPTKYPTLC). We employed a chimeric sequence peptide as immunogen for NAPE-PLD antibody generation. The aim of this chimeric construction was to obtain two distant epitopes exposed in the native protein because one of them belongs to the N-terminal and the other to the C-terminal region of the protein, both regions having random coil structures. NAPE-PLD, DAGL alpha and DAGL beta peptides were synthesized and coupled to keyhole limpet hemocyanin (KLH, JPT Peptide Technologies, Berlin, Germany). The three peptides were injected to rabbits (two animals per peptides), according to standard protocols for generation of antisera, with the IgG fraction subsequently purified by means of a protein A column (Sigma, St. Louis, MO, USA).
(0.03 MB DOC)
