# Supplementary material for: Ulcerative Colitis Induces Changes on the Expression of the Endocannabinoid System in the Human Colonic Tissue
Source: PLoS One. 2009 Sep 4;4(9):e6893. doi: 10.1371/journal.pone.0006893 (PMC2731878; doi:10.1371/journal.pone.0006893)
Supplement: Supporting Information S1 — Generation of NAPE-PLD-, DAGLα-, DAGLβ-specific antibodies. We have generated polyclonal rabbit antibodies against proteins of the cannabinoid machinery. Immunizing peptides were 1) a 13-amino-acid (aa) peptide comprising part of both the C-terminal and the N-terminal regions of NAPE-PLD (MDENSCDKAFEET); 2) a 16-aa peptide from the C-terminal region of DAGL alpha (CGASPTKQDDLVISAR); 3) a 16-aa peptide from an internal sequence of DAGL beta (SSDSPLDSPTKYPTLC). We employed a chimeric sequence peptide as immunogen for NAPE-PLD antibody generation. The aim of this chimeric construction was to obtain two distant epitopes exposed in the native protein because one of them belongs to the N-terminal and the other to the C-terminal region of the protein, both regions having random coil structures. NAPE-PLD, DAGL alpha and DAGL beta peptides were synthesized and coupled to keyhole limpet hemocyanin (KLH, JPT Peptide Technologies, Berlin, Germany). The three peptides were injected to rabbits (two animals per peptides), according to standard protocols for generation of antisera, with the IgG fraction subsequently purified by means of a protein A column (Sigma, St. Louis, MO, USA). (0.03 MB DOC) [file pone.0006893.s001.doc]

**SUPPORTING INFORMATION**

**Generation of NAPE-PLD-, DAGLα-, DAGLβ-specific antibodies**

We have generated polyclonal rabbit antibodies against proteins of the cannabinoid machinery. Immunizing peptides were 1) a 13-amino-acid (aa) peptide comprising part of both the C-terminal and the N-terminal regions of NAPE-PLD (MDENSCDKAFEET); 2) a 16-aa peptide from the C-terminal region of DAGLα (CGASPTKQDDLVISAR); 3) a 16-aa peptide from an internal sequence of DAGLβ (SSDSPLDSPTKYPTLC). We employed a chimeric sequence peptide as immunogen for NAPE-PLD antibody generation. The aim of this chimeric construction was to obtain two distant epitopes exposed in the native protein because one of them belongs to the N-terminal and the other to the C-terminal region of the protein, both regions having random coil structures. NAPE-PLD, DAGLα and DAGLβ peptides were synthesized and coupled to keyhole limpet hemocyanin (KLH, JPT Peptide Technologies, Berlin, Germany). The three peptides were injected to rabbits (two animals per peptides), according to standard protocols for generation of antisera, with the IgG fraction subsequently purified by means of a protein A column (Sigma, St. Louis, MO, USA).
